# Investigative Study on Nitric Oxide Production in Human Dermal Fibroblast Cells under Normal and High Glucose Conditions

**DOI:** 10.3390/medsci6040099

**Published:** 2018-11-09

**Authors:** Maria P. Kwesiga, Emily Cook, Jennifer Hannon, Sarah Wayward, Caroline Gwaltney, Smitha Rao, Megan C. Frost

**Affiliations:** 1 Department of Biomedical Engineering, Michigan Technological University, Houghton, MI 49931, USA; mpkwesig@mtu.edu (M.P.K.); jnhannon@mtu.edu (J.H.); sawaywar@mtu.edu (S.W.); smithar@mtu.edu (S.R.); 2 Doctoral Program in Physical Therapy, Central Michigan University, Mt. Pleasant, MI 48859, USA; cook2ek@cmich.edu (E.C.); gwalt1c@cmich.edu (C.G.)

**Keywords:** diabetic foot ulcers, fibroblast cells, real-time nitric oxide, nitrite

## Abstract

Diabetic foot ulcers (DFU) are a major health problem associated with diabetes mellitus. Impaired nitric oxide (NO) production has been shown to be a major contributor to the dysregulation of healing in DFU. The level of impairment is not known primarily due to challenges with measuring NO. Herein, we report the actual level of NO produced by human dermal fibroblasts cultured under normal and high glucose conditions. Fibroblasts produce the extracellular matrix, which facilitate the migration of keratinocytes to close wounds. The results show that NO production was significantly higher in normal glucose compared to high glucose conditions. The real-time NO detected was compared to the nitrite present in the culture media and there was a direct correlation between real-time NO and nitrite in normal glucose conditions. However, real-time NO detection and nitrite measurement did not correlate under high glucose conditions. The inducible nitric oxide synthase (iNOS) enzyme responsible for NO production was upregulated in normal and high glucose conditions and the proliferation rate of fibroblasts was not statistically different in all the treatment groups. Relying only on nitrite to assess NO production is not an accurate determinant of the NO present in the wound bed in pathological states such as diabetes mellitus.

## 1. Introduction

Diabetes mellitus (DM) was targeted as one of the major non-communicable diseases (NCDs) that threaten development in the 21st century at the 2011 United Nations High-level Political Meeting [1,2,3]. In 2012, DM was directly responsible for 1.5 million deaths worldwide and as of 2017, the global prevalence of DM has risen to 425 million people [4,5]. Annually, 9.1 to 26.1 million of the population affected with DM develop a diabetic foot ulcer (DFU) [6]. The risk of death of a patient with a DFU is 2.5 times higher compared to a patient without a foot ulcer [6,7]. In another study, the mortality at 5 years was reported to be 45 and 55 percent in diabetic patients with neuropathic and ischemic ulcers respectively [8]. Neuropathy and microcirculatory deficiencies increase susceptibility to infections, which is the major cause of amputations [9]. Wound closure in DFU takes approximately 90 to 120 days, leading to high costs in podiatric care that amounts to at least one-third of the $176 billion spent on the treatment of DM every year in the United States [6,10]. In addition to the financial burden, having a DFU has a negative impact on a patient’s lifestyle, including daily mobility and activity, higher risk of depression and so forth. [10,11]. 

Wound healing is a complex, cell-mediated symphony of events, progressing through a series of predictable and overlapping stages: hemostasis, inflammation, proliferation and remodeling [9]. During hemostasis, platelets adhere and aggregate at the wound site to prevent blood loss. The inflammatory phase is characterized by the migration of neutrophils and monocytes. The monocytes differentiate into macrophages to fight off infection and clean the wound of cellular debris. In the proliferation phase, fibroblast cells proliferate and produce extracellular matrix, which aids in the migration, proliferation and differentiation of keratinocyte cells that are involved in re-epithelialization [9,12]. Fibroblasts also produce growth factors, which aid in angiogenesis and provide the chemical cues for the migration and proliferation of keratinocyte cells [13]. However, in diabetic ulcers, the fibroblasts have been shown to be senescent with a reduced proliferative response to growth factors, implicating them in slower healing [9,12,13,14]. 

More recently, the role of nitric oxide (NO) in wound repair has been reported and there is growing evidence that suggests the production of NO by fibroblasts may play a critical role in signaling during fibroblast proliferation and function [15,16,17,18]. Nitric oxide is a potent signaling mediator in many cellular processes and has been reported to be both beneficial and pathological in the same cell populations when delivered at different doses and for different durations [19,20,21]. For instance, high levels of NO can cause senescence, cell cycle arrest and apoptosis while low levels are associated with intercellular signaling, prevention of platelet aggregation, increased vascular permeability and angiogenesis [19,21]. It has been shown that the inappropriate production of NO is a major contributor to the dysregulation of wound healing [16,22]. Schaffer et al. showed that NO production was suppressed in wound cells isolated from diabetic mice and the production of NO was restored by treatment with insulin [17]. Also, collagen deposition and wound mechanical strength was improved with an increase in the NO levels. The same group reported that wound fibroblast cells produce NO while non-activated fibroblasts do not produce NO. Furthermore, the production of NO was reduced in subsequent cell passages [17]. In contrast, one study argues that NO is spontaneously produced from non-activated fibroblast cells with and without stimulation by inflammatory cytokines [18]. 

The difficulty with understanding and harnessing the power of NO in wound healing is that this highly reactive molecule is very challenging to directly measure in physiological conditions because of its very short half-life and its rapid reaction with species present in the wound or cell culture environment [22,23,24]. The generally accepted technique that has been used to assess NO production has been to measure the total accumulation of nitrite using the Griess assay. The assumption is that all the NO produced or delivered to the cells is converted to nitrite and nitrate. Nitrate is reduced to nitrite in the presence of nitrate reductase. The accumulated nitrite will then react with sulfanilamide and N-1-napthylethylenediamine producing an azo dye that can be measured spectrophotometrically [23,25,26]. While this method of indirectly determining NO that was present in the cell samples has its merits, it is not quantitatively accurate. In the study by Hunter et al., the measured nitrite in DMEM media was lower than the theoretical amount of the NO donor N-diazeniumdiolated L-proline (PROLI/NO) and this was attributed to the possible reaction of NO with proteins and other compounds present in the cell culture media [23]. In addition, the detection limit of the Griess assay is approximately 0.1–0.5 µM [23,26,27]. This makes it very challenging to get consistent results for samples containing lower levels of NO. Also, importantly, this is an accumulation assay and there is no means to determine the rate of production of NO or how this rate changes over time. 

Our lab has developed a novel measurement system (the CellNO trap) that allows the direct, real-time, continuous measurement of NO produced or exposed to the cells under conventional culturing conditions using chemiluminescence, one of the most sensitive and specific methods for detecting NO through its reaction with ozone [23,26,27]. This device has previously been validated and used to characterize the rate and amount of NO produced in macrophage cells as well as the actual levels of NO that cells are exposed to when treated with soluble NO donors [28,29]. In this study, we used this tool to specifically elucidate the temporal aspects (both rate and amount) of NO produced by human dermal fibroblasts under normal (5.5 mM) and high glucose (25 mM) conditions and for the first time, we report the actual levels of NO that human dermal fibroblast cells produced in real time. The rate of cell proliferation and the expression of the inducible nitric oxide synthase (iNOS) enzyme responsible for the production of NO when the cells are stimulated by inflammatory cytokines was also assessed. The real- time NO and nitrite accumulation were measured in the cells and cell culture media, respectively and compared. By understanding the levels of NO produced and how the proliferation rate and the protein expression in these cells change under both normal and diabetic conditions, we will begin to clearly understand the levels and timing of NO delivery necessary to promote appropriate fibroblast proliferation, migration and deposition of the extracellular matrix in order to reduce the healing time in chronic DFUs.

## 2. Materials and Methods

### 2.1. Cell Culture and Chemical Supplies

Primary human adult dermal fibroblasts (HDFa, ATCC^®^ PCS-201-012™), mouse macrophages (RAW 264.7), penicillin streptomycin, fetal bovine serum (FBS), phosphate buffered saline (PBS), 3-(4,5-dimethylthiazol-2-yl)-2,5-diphenyltetrazolium bromide (MTT) proliferation assay kit and Dulbecco’s modified eagle medium (DMEM high glucose 4500 mg/L) were all purchased from ATCC (Manassas, VA, USA). DMEM (no glucose), sodium pyruvate and Hoescht dye were obtained from Fischersci (Hanover Park, IL, USA). Lipopolysaccharide *pseudomonas aeroguinosa* (LPS), Calcein-AM, protease inhibitor cocktail, mouse nitric oxide synthase and dopamine-HCl were purchased from Sigma-Aldrich (St. Louis, MO, USA). Ethidium bromide was acquired from Invitrogen (Grand Island, NY, USA). Human recombinant Interferon γ (hrIFN-γ) was purchased from GenScript (Piscataway, NJ, USA). The primary and secondary antibodies against CD90/Thy1 (ab23894) protein and (iNOS) (ab136918) were purchased from Abcam (Cambridge, MA, USA). The 7.5% SDS polyacrylamide gels and gelatin were acquired from Bio-Rad (Hercules, CA, USA). The Odyssey western blot starter kit 2 was obtained from *LI-COR* (Lincoln, NE, USA). Silicone elastomer base and curing agent (Dow Corning Sylgard^®^184) were obtained from ML Solar LLC (Campbell, CA, USA).

### 2.2. Real Time NO Measurements from Primary Human Adult Dermal Fibroblasts

Primary adult dermal fibroblasts at passage 1 were cultured and expanded in conventional cell culture plates (100 mm) in DMEM (high or normal glucose levels), 10% FBS and 1% penicillin/streptomycin (complete growth media). At confluency, the cells were reseeded in CellNO trap devices at a density of 0.25–1 × 10^4^ cells/cm^2^. Previously, we have reported the detailed fabrication and characterization of the device [28]. Briefly, silicone elastomer base and curing agent were dissolved in hexanes, manually cast onto 5 × 5 cm square polyvinylidene fluoride (PVDF) membranes and left to cure in a 50 °C oven for 24 h. The modified membrane was cut into a circle to fit into a 60 mm diameter cell culture plate with the bottom removed forming the upper chamber. The upper chamber was sealed by applying toluene to the edges of the membrane and the plate. A second 60 mm plate with drilled holes and plastic outlets attached was affixed to the upper chamber using toluene and reinforced with epoxy adhesive. The upper chamber was used for cell culture and the lower chamber was used for gaseous transfer. To make the device suitable for cell culture, freshly prepared dopamine solution in Tris-HCl buffer (2 mg/mL) was added to the device and incubated for 12 h. The device was rinsed several times and sterilized using 70% ethanol and ultraviolet light (1 h). Prior to cell culture, the device was coated with gelatin solution (2 mg/mL). After 24–72 h, the cell culture media was changed and substituted with media containing 40 µg/mL of LPS and 200 U/mL of hrIFN-Ƴ or complete growth media (control) under normal glucose (5.5 mM) and high glucose (25 mM) conditions. The device was placed in a standard incubator (37 °C, 65% humidity, 5% CO_2_) and was connected to a calibrated nitric oxide analyzer (NOA) and the real-time NO release profile was measured for 24 h.

### 2.3. Cell Viability Assay in the CellNO Trap Device

After 24 h, the live-dead assay was carried out using 2 µM calcein AM, 2 µg/mL ethidium bromide and 10 µg/mL Hoescht dye in DMEM media for 10–15 min. The cells were imaged and analyzed using an Olympus fluorescent microscope (model BX51) and Image J1 respectively [30].

### 2.4. Cell Proliferation Assays (MTT Assay)

The fibroblast cells were cultured in 96 well plates at a density of 10,000 cells per well in normal and high glucose cell culture conditions and stimulated with 40 µg/mL and 200 U/mL of LPS and IFN respectively. At the end of 24, 48 and 72 h, MTT assay was performed according to the manufacturer’s instructions (Trevigen, 4890-025-K, Gaithersburg, MD, USA) with minor modifications. Briefly, the cell culture media was replaced with 25 µL of the MTT reagent and incubated for 4 h to allow the intracellular reduction of soluble yellow MTT to insoluble formazan dye. The MTT reagent was discarded and 100 µL of Isopropyl alcohol was added to solubilize the dye. The absorbance was read at a wave length of 570 nm using a VERSAmax tunable microplate reader model (Molecular devices, Sunnyvale, CA, USA).

### 2.5. Nitrite Assay

After 24 h of measurement in the CellNO trap device, the cell culture media from the cell samples under normal and high glucose conditions were collected. The triiodide assay was used to measure the nitrite accumulation in the media [26]. Briefly, 50 µL of the media was added to a vial containing a stirring solution of 300 µL of glacial acetic acid and 120 µL of potassium iodide. The vial was connected to a calibrated Sievers Nitric Oxide Analyzer 280i (Zysense, LLC, Boulder, CO, USA) and the NO produced from the nitrite present in the media was measured and normalized to the number of live cells.

### 2.6. Cell Characterization

Fibroblast cells at passage 2 were cultured in 12 well plates at a cell density of 1 × 10^4^/cm^2^ in normal and high glucose conditions. At confluency, the cells were fixed in 4% paraformaldehyde for 10 min, rinsed three times in phosphate buffered saline, incubated in blocking buffer for 90 min followed by the primary antibody for 18 h at 4 °C. The cells were rinsed three times in blocking buffer and incubated for 1 h at room temperature with the secondary antibody, stained with 4’, 6-diamidino-2-phenylindole (DAPI) and viewed under a fluorescent microscope. 

### 2.7. Western Blot Analysis

Confluent HDFa cells were cultured in 100 mm diameter tissue culture dishes, stimulated with 40 µg/mL of LPS and 200 U/mL IFN-γ in normal and high glucose conditions and incubated for 72 h. Mouse macrophage cells RAW 264.7 were grown to confluency and stimulated with 100 ng/mL of LPS for 18 h and served as the positive control. The cells were quickly washed in ice cold PBS, trypsinized, centrifuged and re-suspended in 200 µL of RIPA buffer in the presence of protease cocktail inhibitors. The cell lysates were incubated for 30 min on ice on a plate shaker and centrifuged at 12,000 rpm for 20 min at 4 °C. The supernatant was collected and the protein concentration determined for each sample using Bradford assay [31]. An equal amount of loading buffer containing dithiothreitol was added to each sample, boiled at 100 °C for 5 min and immediately placed on ice for 2 min. About 5.63–9.37 µg of protein was loaded in a 7.5% SDS-PAGE gel and separated by electrophoresis at 100 V for approximately 70 min. The proteins were transferred to immobilon^®^-FL PVDF membrane using the Trans-Blot^®^ Turbo™ Transfer System (Bio-Rad) for 7 min. The membrane was incubated in Odyssey blocking buffer (TBS) for 60 min to block nonspecific binding sites followed by incubation in TBS containing 0.2% of tween20 (TBS-T) and the iNOS antibody (1:1000 dilution) for 18 h at 4 °C. After that, the membrane was washed 4 times with TBS (0.1% tween20) and incubated in TBS-T with the secondary antibody (1:20,000 dilution) for 1 h then extensively washed in TBS, visualized by *LI-COR* Odyssey infrared imager and analyzed using Image Studio Lite software Version 5.2. 

Statistical analysis was performed in Microsoft Excel using one-way ANOVA followed by a Bonferroni corrected *t*-test with statistical significance set at a 95% confidence level (*p* < 0.05). 

## 3. Results

### 3.1. Real-Time NO Detected under Normal and High Glucose Conditions

**Figure 1:**
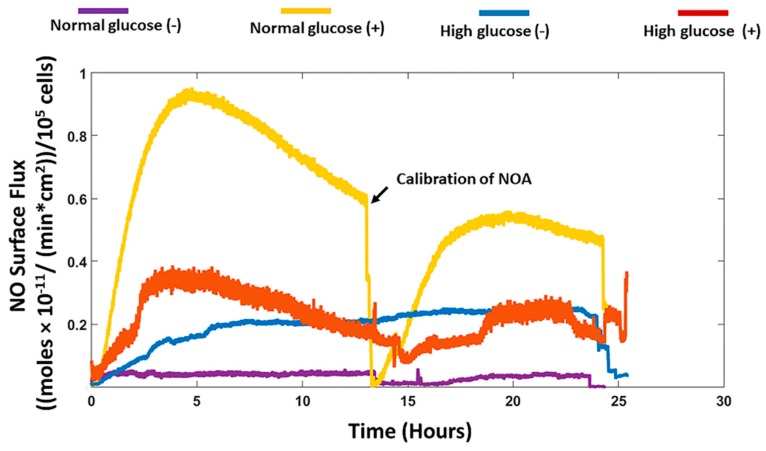
An example of real-time nitric oxide (NO) surface flux (normalized to the cell number) generated from HDFa with (+) and without (−) stimulation cultured under normal (5.5 mM) and high glucose (25 mM) conditions measured with chemiluminescence detection using a CellNO trap.

Primary human adult dermal fibroblasts were cultured in the CellNO trap in either normal (5.5 mM) or high glucose (25 mM) media with and without stimulation by the inflammatory cytokine interferon gamma (IFN-γ) and bacterial endotoxin lipolysaccharide (LPS). The NO production was measured for 24 h (Figure 1). The cells cultured in normal glucose media in the absence of stimulation showed a slight increase in NO release in the first 1 h which peaked at 0.5 × 10^−12^ (moles/min·cm^2^)/10^5^ cells and remained constant for 24 h. The HDFa cultured in high glucose media without stimulation showed an increase in NO production which peaked at 0.19 × 10^−11^ (moles/min·cm^2^)/10^5^ cells and remained constant for 24 h. With stimulation, there was an increase in the NO production for cells cultured in normal glucose media, which peaked at 0.94 × 10^−11^ (moles/min·cm^2^)/10^5^ cells and gradually dropped to 0.6 × 10^−11^ (moles/min·cm^2^)/10^5^ cells. At 13 h, the NOA was disconnected and recalibrated to confirm the validity of the signal obtained (Arrow denoting the sudden drop in curve in Figure 1). The NO detected remained fairly constant around 0.5 × 10^−11^ (moles/min·cm^2^)/10^5^ cells up to 24 h. The NO release for cells cultured in high glucose media followed a similar trend but with lower NO detected, which peaked at 0.35 × 10^−11^ (moles/min·cm^2^)/10^5^ cells at approximately 4 h and gradually dropped to 0.81 × 10^−12^ (moles/min·cm^2^)/10^5^ cells at 15 h with fluctuations below 0.3 × 10^−11^ (moles/min·cm^2^)/10^5^ cells for 24 h. 

**Figure 2:**
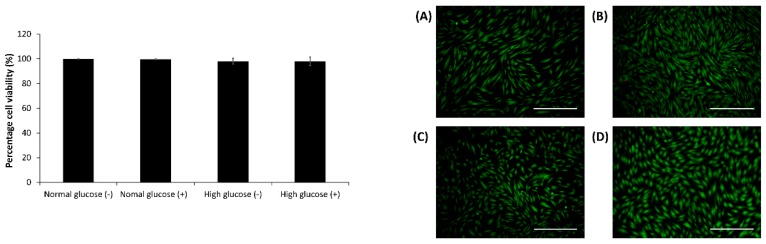
Cell viability detected by calcein AM and ethidium bromide for primary human adult dermal fibroblasts (HDFa) cultured in normal (5.5 mM) glucose condition with and without stimulation, (**A**,**B**) respectively and high glucose (25 mM) condition without and with stimulation (**C**,**D**) respectively. Scale bar 500 µm. The results are presented as the mean ± standard deviation for *n* = 3, * *p* < 0.05.

The viability of the cells cultured in the CellNO trap device in normal and high glucose culture conditions with and without stimulation by IFN-γ and LPS was analyzed by the live/dead assay at the end of the real-time NO measurements. The cells maintained over 95% cell viability in all the treatment groups (Figure 2).

### 3.2. Nitric Oxide Detected from Nitrite Accumulation in the Absence and Presence of Stimulation

**Figure 3:**
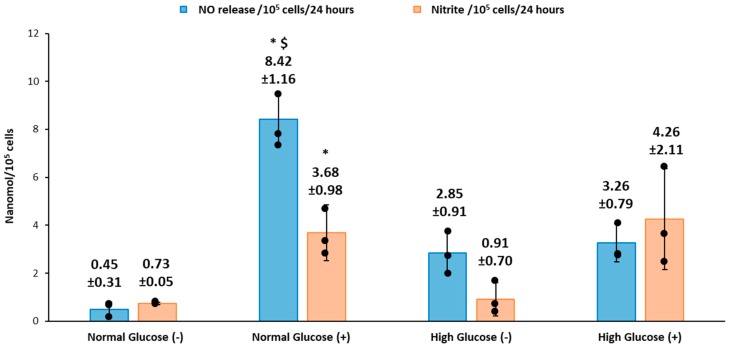
Total NO compared to nitrite accumulation determined for HDFa with and without stimulation under normal and high glucose conditions. (For *n* = 3, there is statistically significant difference at *p* < 0.05: * Normal glucose (LPS/IFN) vs. Normal glucose (without stimulation); $ Normal glucose (LPS/IFN) versus High glucose (LPS/IFN), dots on each average bar indicate individual data points.

After the real-time NO measurements, the cell culture media was collected and analyzed for nitrite using the triiodide assay (Figure 3). The nitrite present in the media is reduced in the presence of an acid and nucleophile to produce NO that is measured via chemiluminescence detection [26]. Normal glucose conditions without stimulation had significantly lower NO levels produced from nitrite compared to normal glucose conditions with stimulation (0.73 ± 0.05 versus 3.68 ± 0.98 nmol/10^5^ cells, *p* < 0.05 for *n* = 3). In high glucose conditions, there was no statistical difference in the levels of NO detected without stimulation compared to stimulation with LPS/IFN (0.91 ± 0.70 versus 4.26 ± 2.11 nmol/10^5^ cells). In the absence of stimulation, the NO detected from nitrite in normal glucose conditions compared to high glucose were not significantly different (0.73 ± 0.05 versus 0.91 ± 0.70 nmol/10^5^ cells). Similarly, with stimulation, the NO detected from nitrite was not statistically different in normal glucose compared to high glucose (3.68 ± 0.98 versus 4.26 ± 2.11 nmol/10^5^ cells).

### 3.3. Comparison of Real-Time NO Measurements and Nitrite Accumulation

The total NO detected in real-time for the HDFa was determined by integrating the area under the curve (Figure 1) and was compared to the NO inferred from nitrite present in the cell culture media for the 4 treatments groups (Figure 3). The results showed that the levels of real-time NO detected in nmol/10^5^ was significantly higher for cells in normal glucose conditions with stimulation (8.42 ± 1.16 *p* < 0.05 for *n* = 3) compared to without stimulation (0.48 ± 0.31 for *n* = 3 *p* < 0.05). Similarly, under identical conditions, nitrite accumulation was significantly higher in normal glucose with stimulation (3.68 ± 0.98) compared to absence of stimulation (0.73 ± 0.05 for *n* = 3 *p* < 0.05). It is interesting to note that there was a higher direct NO measured compared to nitrite. However, high glucose conditions did not show the same trend. The levels of real-time NO detected with stimulation were not statistically different from levels of real-time NO without stimulation (3.26 ± 0.79 and 2.85 ± 0.91 nmol/10^5^ cells respectively).The NO levels obtained from nitrite measurement depicted a similar trend as the real-time NO measured, with no statistical difference in high glucose conditions with stimulation compared to high glucose without stimulation (4.26 ± 2.11 and 0.91 ± 0.70 nmol/10^5^ cells). The real-time levels of NO and nitrite detected in normal glucose conditions without stimulation were also not statistically different from the real time NO detected from the cells in high glucose conditions without stimulation (0.48 ± 0.31 and 3.26 ± 0.79 versus 0.73 ± 0.05 and 0.91 ± 0.70 nmol/10^5^ cells respectively for *n* = 3 *p* < 0.05). Moreover, in normal glucose conditions with stimulation, the levels of real-time NO detected was significantly higher compared to high glucose conditions with stimulation. The nitrite levels were not statistically different. (8.42 ± 1.16 and 3.26 ± 0.79 versus 3.68 ± 0.98 and 4.26 ± 2.11 nmol/10^5^ cells respectively).

### 3.4. Cell Characterization of HDFa Cultured in Normal and High Glucose Conditions

**Figure 4:**
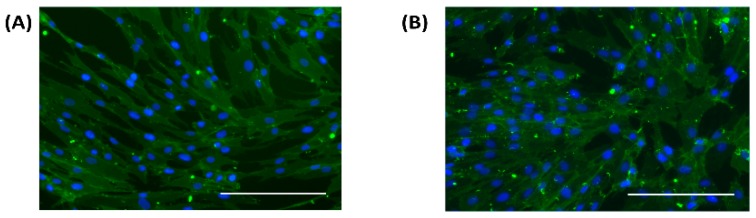
Immunofluorescent staining of CD90 (Thy-1) (green) in HDFa cultured in (**A**) Normal and (**B**) High glucose conditions. Cell nuclei (blue). Scale bar 200 µm.

Primary human adult dermal fibroblasts cultured in normal and high glucose media were assessed for the presence of CD90 (Thy-1), which is a reliable molecular marker used to identify fibroblast cells in culture [32]. The HDFa maintained uniform staining for CD90 (Thy-1) protein in normal (Figure 4a) and high glucose conditions (Figure 4b) as well as the elongated and spindle shape that is characteristic of confluent fibroblast cells.

### 3.5. Expression of iNOS Protein in HDFa Cultured in Normal and High Glucose Conditions

**Figure 5:**
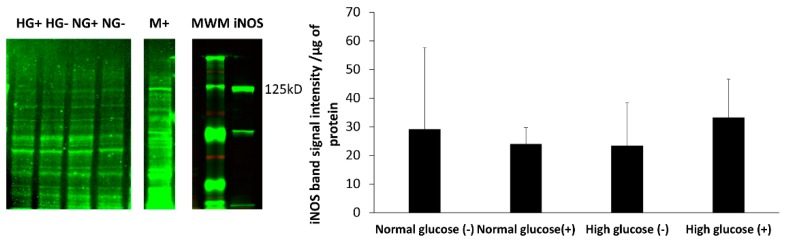
Detection of iNOS expression by western blot analysis of HDFa cultured in normal (NG) and high glucose (HG) conditions with (+) and without (−) stimulation. Predicted band size for iNOS 131 kD and observed band size 125kD with iNOS and RAW 264.7 (M+) as positive controls. MWM—Molecular weight marker.

Stimulation of fibroblasts by inflammatory cytokines and bacterial endotoxins has been reported to upregulate the enzyme iNOS, which is directly responsible for the production of NO in the cells. The levels of iNOS in HDFa were assessed in cells cultured in normal and high glucose conditions with and without stimulation by western blot. The results show that overall the iNOS enzyme was present in all treatment groups and the protein level was very low. Macrophage cell lysates (RAW 264.7) that are known to express iNOS when stimulated with LPS and the mouse iNOS enzyme protein were used as positive controls. Faint iNOS bands can be seen in the human fibroblasts cells, which appear at the same molecular weight as the iNOS in macrophage cells and the mouse iNOS enzyme (Figure 5). There was no statistical difference in intensity found between the groups at *p* < 0.05 for *n* = 3.

### 3.6. Effect of Normal and High Glucose Conditions with and without Stimulation on the Proliferation of HDFa Cells

**Figure 6:**
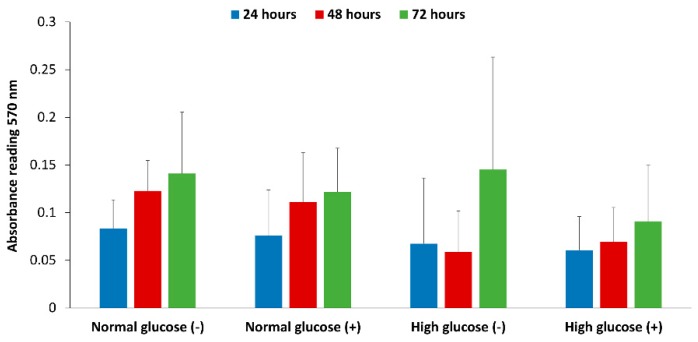
The rate of proliferation observed in HDFa cultured in normal (5.5 mM) and high glucose (25 mM) conditions with (+) and without (−) stimulation from 24 to 72 h. Three independent experiments were performed in triplicates (*n* = 3). *p* value < 0.05 was considered statistically significant.

The proliferation rate of HDFa cells cultured in normal and high glucose conditions with and without stimulation by IFN-γ and LPS was assessed by the MTT proliferation assay for 24 to 72 h. The cells that are metabolically active reduce the tetrazolium salts in the MTT reagent to intracellular purple formazan crystals, which when solubilized can be read at an absorbance of 570 nm and correlated to the proliferation of the cells [33]. In normal glucose conditions in the absence and presence of stimulation there was an increase in cell proliferation from 24 to 72 h (0.08 to 0.14 and 0.08 to 0.12 respectively). High glucose conditions with no stimulation showed a slight drop in absorbance from 24 to 48 h (0.07 to 0.06) and an increase to 0.15 at 72 h. High glucose conditions with stimulation also showed an increase in absorbance; 0.06 at 24 h to 0.09 at 72 h. Overall, there was no statistical difference between the treatment groups at 24, 48 and 72 h (*n* = 3 *p* < 0.05) (Figure 6).

## 4. Discussion

Diabetic foot ulcers remain a growing problem with debilitating consequences and no adequate treatment. One of the major problems in the delayed healing of these wounds is the senescent nature of fibroblasts and their inability to proliferate and produce extracellular matrix that will aid in the migration of keratinocytes across the wound bed [9,12,13,14,34]. Nitric oxide is widely implicated as a major player in the wound healing process and studies have shown that fibroblast cells produce NO [15,17,18,22]. Whether or not this NO production is consistent even in the absence of stimulation is still a matter of debate. Moreover, the spatial and temporal NO production and levels that fibroblast cells experience is not known. The Griess assay used by previous authors only looks at the accumulated nitrite levels (the stable end product of NO decomposition and not NO itself) at single point in time and attributes this level to NO production. The availability of NO to regulate biological functions greatly relies on various factors such as the dose, duration, location of NO production as well as the surrounding environment [19,20,21]. Therefore, the presence of nitrite in the cell culture media might not automatically translate to the actual level of NO generated and therefore cast doubt on the causal relationship NO may or may not actually have in the physiological environment [19,21]. Wound healing is a dynamic process and understanding how the levels of NO production in the cells at the wound site changes over time is of utmost importance in exploring the possible use of NO to treat DFUs. 

We recorded the real-time production of NO from fibroblast cells under normal and high glucose conditions with and without stimulation by the bacterial endotoxin LPS and inflammatory cytokine IFN-γ. The results obtained were then compared to the nitrite accumulation in the cell culture media. HDFa cells in normal glucose conditions with stimulation showed that significantly higher NO was detected compared to HDFa cultured in normal glucose conditions without stimulation (8.42 ± 1.16 versus 0.48 ± 0.31 nmol/10^5^cells, *p* < 0.05 for *n* = 3). Stimulation by LPS and IFN-γ is thought to upregulate the expression of iNOS and consequently NO production in a variety of cells including human and rodent fibroblasts [18,35]. In cells such as macrophages, the production of NO acts as a host defense mechanism to fight off infection [22,36]. In the normal wound healing process, the production of NO in fibroblast cells could act to regulate the inflammatory response at the wound site [18]. Alternatively and/or additionally the production of NO could be associated with regulation of cell proliferation, collagen deposition or angiogenesis, which would show its possible role in the proliferation stage [17,18,22]. The NO detected from the cells cultured in high glucose media with stimulation was not significantly different from the NO detected in non-stimulated cells cultured in the same media. Cells cultured in normal glucose conditions in the absence of stimulation showed very low levels of NO compared to high glucose conditions without stimulation although the results were not statistically different (Figure 1 and Figure 3). Schaffer et al. showed that fibroblast cells do not produce NO unless they are phenotypically altered to produce NO (wound fibroblasts) [17]. However, Wang et al. showed that fibroblast cells spontaneously produce NO even in the absence of stimulation [18]. The levels of glucose used in the cell culture media were not specified in Wang’s study. High glucose DMEM media that is routinely used in culture of a variety of mammalian cells contains 25mM of glucose, which is four times greater than clinically relevant glucose levels (5.5–7 mM) [37]. Therefore, the levels of glucose in the cell culture media could possibly explain the discrepancy reported in the literature. Cells cultured in normal glucose conditions with stimulation had significantly higher levels of NO detected compared to stimulated cells cultured in high glucose conditions (Figure 3). Low levels of NO and reduced nitric oxide synthase levels in the diabetic state has been strongly associated with the development of DFU and impaired wound healing [38,39,40]. Schaffer et al. found that low NO levels correlated with reduced collagen deposition and wound strength in wounded diabetic mice compared to healthy mice [17]. The mechanism of low NO levels in the diabetic state is still unclear but evidence points toward the increased production of reactive oxygen species, such as superoxide, that rapidly inactivate NO, reducing its bioavailability in the cells [21,22]. It is worthwhile to note that all of these studies only looked at nitrite accumulated in the cell culture media and not NO levels directly exposed to the surface of the cells. Our work has shown that the NO and nitrite levels are different under normal and high glucose conditions and the variability is further compounded when stimulated by NO inducing factors such as LPS and IFN-γ. The real-time NO measurements were compared to the nitrite accumulated in the cell culture media. The levels of real-time NO in normal glucose conditions directly correlated with the nitrite levels in the presence and absence of stimulation. However, high glucose conditions showed a discrepancy in real-time NO and nitrite levels detected (in both cases, the results were not statistically different, but the difference observed suggests further study is warranted). Human aortic endothelial cells cultured in high glucose media has been shown to have higher levels of nitrite compared to cells cultured in normal glucose conditions in the absence of stimulation [41]. In another study, nitrite levels were suppressed in human renal endothelial and mouse Schwann cells cultured in high glucose media compared to normal glucose media [42,43]. The contradicting results obtained from these studies further illustrates that measuring only nitrite in the media is not sufficient to assess actual NO production and thereby attribute specific effects of NO on different cellular processes especially under a pathological state such as diabetes. 

The iNOS enzyme is thought to be upregulated when cells are stimulated by inflammatory mediators. We analyzed the expression of iNOS in fibroblast cells in normal and high glucose levels with and without stimulation by LPS and IFN-γ. The iNOS enzyme was found in both normal and high glucose conditions in the absence and presence of stimulation. Although, the expression was very low with no statistical difference between the four treatment groups. Fibroblast cells produce low amounts of NO, which could explain why the enzyme expression in the cells was low. To support this view, Wang showed that with immunocytochemical staining, only few stimulated fibroblast cells stained intensely for iNOS, while majority of the cells showed weak staining. Some unstimulated fibroblast cells also showed weak expression of iNOS. Interestingly, histological staining on the dermal tissue was negative for iNOS [18]. There are various factors in the cell culture system that could potentially affect cell response. An example of rat hepatocytes was reported by Halliwell; Isolation of the cells from the tissue was found to activate iNOS in the absence of stimulation. The author also reported that the oxygen levels in in vitro cell culture systems (150 mmHg) was found to be much higher than the physiological levels of oxygen that the cells are exposed to in vivo (1–10 mmHg) [44]. These high levels of oxygen are associated with increased production of reactive oxygen species (ROS). Reactive oxygen species activate the Nuclear factor-kappa B (NF-KB) signaling pathway that leads to transcription of iNOS mRNA and increased expression of the iNOS enzyme [45]. Moreover, ROS such as superoxide rapidly react with NO forming peroxynitrite. The NO deficiency in the cell environment is hypothesized to activate a feedback mechanism that upregulates iNOS expression [46]. In regard to these findings, we speculate that other factors including the levels of glucose present in the cell culture environment might explain the differences of nitrite levels compared to the real-time levels of NO observed. 

Nitric oxide has been shown to regulate the proliferation of cells and production of extracellular matrix [47,48]. In the diabetic wound, fibroblast cells do not proliferate adequately and there is reduced secretion of extracellular matrix, which is a key component for the migration and differentiation of keratinocyte cells [9,12,16]. Xuan et al. have explored the effect of high glucose on human foreskin fibroblast proliferation with no significant difference at up to 60 mM of glucose concentration (media supplemented with 10% FBS) [49]. In Schaffer’s study inhibition of endogenous iNOS production in mouse wound fibroblasts did not affect proliferation [17]. While Shi et al. showed reduced proliferation of dermal fibroblast cells isolated from iNOS knock out mice compared to wild type mice [50]. The correlation between glucose levels in the presence of inflammatory mediators that stimulate NO production and its effect on human dermal fibroblast cell proliferation has not been previously reported. We looked at the effect of normal and high glucose conditions with and without stimulation of NO production on the proliferation of fibroblast cells. The results show that overall there was no statistical difference between normal and high glucose conditions in the absence and presence of stimulation from 24 to 72 h (Figure 6). However, the proliferation was lower in high glucose condition with stimulation at 48 and 72 h compared to normal glucose with and without stimulation at the same time points, which prompts further investigation.

To conclude, our study showed that human dermal fibroblast cells cultured in normal glucose conditions with stimulation show significantly higher levels of NO compared to cells cultured in high glucose conditions with stimulation (8.42 ± 1.16 and 3.26 ± 0.79 nmols/10^5^ cells *p* < 0.05 for *n* = 3). The nitrite levels in normal glucose conditions correlated with the real-time NO levels in normal glucose conditions in the absence and presence of stimulation. In contrast, there was no significant difference between the real-time NO measured and the nitrite levels that accumulated in the media in high glucose conditions with and without stimulation (Figure 3). The nitrite level in the media is not a reliable proxy for the actual level of NO produced by the cell. The iNOS enzyme that produces NO when cells are stimulated with inflammatory mediators was expressed at a low level in all the treatment groups and the proliferation of fibroblast cells from 24 to 72 h showed no statistical difference between normal and high glucose conditions in the absence or presence of LPS and INF-γ (Figure 5 and Figure 6 respectively). The results presented here clearly illustrate why nitrite accumulation should not be used to predict NO production in cells and clearly indicates that real-time NO measurement is essential in providing information that will allow a more complete understanding of how NO may affect the proliferation, migration and formation of extracellular matrix in fibroblast cells as well as the NO production in other cells present at the wound site. If we accurately measure the actual levels of NO produced by cells cultured under specific conditions, including the temporal NO release profile, we can understand and mimic the physiological NO release at different stages of wound healing and different pathological states, which will facilitate a smooth transition of the wound from its chronic state into resolution, consequently, shortening the time to complete healing of DFU. Tackling the problem of DFU, a strong predictor associated with increased mortality in DM patients, will address one of the nine targets (mortality) set by WHO member states for the prevention and control of NCDs.
